# How vision shapes the paths we choose

**DOI:** 10.7554/eLife.104965

**Published:** 2024-12-09

**Authors:** Arthur D Kuo

**Affiliations:** 1 Faculty of Kinesiology and Department of Biomedical Engineering, University of Calgary Calgary Canada

**Keywords:** natural locomotion, visual control of locomotion, photogrammetry, visual decisions, locomotion, Human

## Abstract

A mathematical model can predict the path walkers take through a rugged landscape, including the tendency of people to avoid paths that are too steep, even if it means going farther.

**Related research article** Muller KS, Panfili D, Shields S, Matthis JS, Bonnen K, Hayhoe MM. 2024. Foothold selection during locomotion in uneven terrain: Results from the integration of eye tracking, motion capture, and photogrammetry. *eLife* **12**:RP91243. doi: 10.7554/eLife.91243.

When walking, our brain needs to make decisions, such as where to step and which path to follow. Visual inputs are central to this process, as they allow us to continuously estimate our motion and orientation, as well as regulate our direction and speed (Warren et al., 2001).

This continuous regulation can be explained using mathematical models that rely on vector calculus (Matthis et al., 2022). However, walking also involves discrete choices – such as selecting which available foothold to use, or turning left or right – that are guided by visual features of the path ahead, such as the rockiness and steepness of the terrain (Matthis et al., 2018). While it may seem obvious that people avoid rugged paths when easier alternatives exist, there is no mathematical model to explain this behavior. Now, in eLife, Mary Hayhoe and colleagues – including Karl Muller as first author – report the results of a study that sheds new light on how these decisions are made (Muller et al., 2024).

The researchers analyzed data from previous studies in which they had tracked the eye and body movements of nine individuals as they walked across natural terrains (Matthis et al., 2022; Bonnen et al., 2021). Participants also wore eye-tracking cameras that captured visual information about where they were looking within the surrounding physical landscape. Muller et al. used this data to create a three-dimensional computational reconstruction of the terrains, which they aligned to the body and eye movements of the participants. This allowed them to develop egocentric visual depth images (which estimated how the retinas of individuals detect the depth of the terrain), and to determine where participants placed their feet.

The researchers – who are based at the University of Texas at Austin, Indiana University and Northeastern University – then used these visual depth images and foothold locations to train a neural network where to step in the reconstructed terrain. The trained model successfully predicted where the human participants placed their feet better than chance, suggesting that decisions about where to step are influenced by visual depth cues. This agrees with prior work showing reduced depth cues cause walkers to shift their gaze to closer footholds, perhaps so they can devote more attention to resolving depth ambiguities (Bonnen et al., 2021).

Next, Muller et al. used their model to investigate whether walkers chose flatter, more winding paths over direct, steeper alternatives that require more energy to cross due to the uneven terrain (Darici and Kuo, 2023; Kowalsky et al., 2021). The team compared two metrics: tortuosity, which is the ratio between the length of a path and the direct distance between its start and end; and a dimensionless measure of ruggedness, equal to the average angle of the up and down steps within a path. These metrics were recorded for the actual path the walkers took, and also for simulated, alternative routes they might have taken through the same terrain.

Muller et al. found that the tortuosity of the paths chosen by the human walkers correlated with the ruggedness of the most direct simulated path through the terrain: the steeper and more rugged the direct path, the more likely a walker is to detour and take a longer, flatter route. This suggests a trade-off whereby walkers assess paths with high tortuosity to be less energetically costly than ones which are straighter but more rugged. Muller et al. also discovered that this trade-off was stronger for shorter individuals, possibly due to greater difficulty with steep steps, though this effect warrants further study (as discussed in the public reviews of the article).

Studying locomotion in natural settings is challenging, as terrains and visual features are harder to manipulate or measure compared to controlled laboratory environments. The methods created by Muller et al. open up new avenues for understanding decision-making during locomotion. However, there is much about the trade-off proposed in the study that remains unknown.

For example, controlled experiments are needed to confirm whether the ruggedness of the simulated direct paths match those experienced in real life. In addition, the human eye likely perceives other aspects of the terrain beyond just depth, and it remains to be seen whether other visual cues help evaluate different structural properties of the landscape, such as friction and the hardness of footholds. Related issues in natural locomotion, such as energy expenditure, also merit further investigation. For instance, the energetic costs of tortuous versus rugged paths still need quantification, as do temporal costs, which are also suspected to govern ecological behavior (Carlisle and Kuo, 2023).

The approach taken by Muller et al. could also be used to study people in human-designed environments, such as pedestrians crossing busy roads (do people jaywalk rather than use pedestrian crossings to save energy, time, or other costs?). Whether navigating natural landscapes or cityscapes, humans constantly create their own paths according to a variety of physical and perceptual criteria.
